# Influence of Age and Nutritional Status on Flight Performance of the Asian Tiger Mosquito *Aedes albopictus* (Diptera: Culicidae)

**DOI:** 10.3390/insects4030404

**Published:** 2013-07-26

**Authors:** Christian Kaufmann, Lauren F. Collins, Mark R. Brown

**Affiliations:** 1 Department of Entomology, University of Georgia, Athens, GA 30602, USA; E-Mails: lfkelly@bu.edu (L.F.C.); mrbrown@uga.edu (M.R.B.); 2 Swiss National Centre for Vector Entomology, Institute of Parasitology, University of Zürich, CH-8057 Zürich, Switzerland

**Keywords:** *Aedes albopictus*, flight potential, distance, vector, mosquito

## Abstract

The Asian tiger mosquito, *Aedes albopictus*, is a competent vector for arboviruses and recently was implicated as the vector of the first autochthonous cases of dengue and chikungunya in southern Europe. The objective of this study was to analyze the flight performance of female *Ae. albopictus* of different ages that were starved, sugar-fed, or sugar-fed and blood-fed, using flight mills. After three days of starvation post emergence, females flew an average distance of 0.7 ± 0.5 km in 1.9 ± 1.5 h during a 16 h trial period, whereas sugar- or sugar- and blood-fed females of this age covered a significantly higher distance of around 3 km with a mean total flight time of around 6 h. The age of females (up to four weeks) had no effect on performance. The average of maximal continuous flight segments of sugar-fed (2.14 ± 0.69 h) and blood-fed (3.17 ± 0.82 h) females was distinctly higher than of starved females (0.38 ± 0.15 h) of which most flyers (83%) performed maximal flight segments that lasted no longer than 0.5 h. Overall, the results for the laboratory monitored flight performance of *Ae. albopictus* confirm their ability to disperse a few kilometres between breeding site and host.

## 1. Introduction

The Asian tiger mosquito *Aedes albopictus* (Skuse) has spread from Southeast Asia to Europe, the Middle East, Africa and the Americas over the past 30 years. This remarkable expansion was aided by the global trade of goods, especially scrap tires [1] and plants like lucky bamboo [2], carrying mosquito eggs or larvae. In Europe, this species was first identified in Albania in 1979 [3] and is now widely established in southern Europe [4,5,6]. In 1985, *Ae. albopictus* was found in Texas, USA [7], and by the 1990s, it had nearly displaced the yellow fever mosquito, *Ae. aegypti*, across the southeastern U.S., although in a few areas both species coexist [8]. *Aedes albopictus* is a competent vector to transmit dengue virus, chikungunya virus, and other arboviruses to humans [8]. To date, it has not been implicated in dengue or chikungunya epidemics in the Americas, but it was the evident vector in recent outbreaks of chikungunya fever on islands of the Indian Ocean and in Italy [9,10,11]. In southern France, two independent cases of autochthonous dengue and two of chikungunya fever that occurred in the autumn of 2010 were transmitted by this species [12]. Arboviruses are disseminated widely by travelling infected humans [13,14], but their local transmission is dependent on the availability of susceptible mosquito species such as *Ae. albopictus* [15]. The flight performance of susceptible female mosquitoes is an important determinant of their host-seeking ability, dispersal potential, and longevity [16], all of which affect vectorial capacity. Laboratory experiments using flight mills are one option to analyze flight performances, and this approach has been pursued in past studies [16,17,18,19]. More recently, differences between the flight performance of wild type and transgenic males of *Ae. aegypti* were obtained with flight mills [20]. The goals of our study were to determine the effects of aging and nutritive states on the flight performance of female *Ae. albopictus*, thus providing insight into the potential dispersal capability of this important arbovirus vector and its displacement of *Ae. aegypti* in the U.S.

## 2. Material and Methods

**Mosquitoes:** The colony of *Ae. albopictus* (originally collected by Prof. Hans Briegel in Louisiana, U.S. and maintained as a laboratory strain over 20 years) was held at 26 ± 1 °C and 85 ± 10% relative humidity under long-day conditions (16 h light, 8 h dark). For egg production, females were allowed to feed on an anesthetized rat. After hatching, first instar larvae were transferred to trays (34 × 21 × 5 cm; 200–250 larvae/tray) containing 700 mL distilled water and fed a daily regimen of pulverized Tetramin^®^ [21] to support optimal growth and development. Pupae were collected into shallow cups that were placed into plexiglas and wire-screen walled cages (24 × 19 × 18.5 cm) to contain emerging adults (200–300/cage). Adults had continuous access to distilled water (starved) or to 10% fructose solution (sugar-fed), depending on experimental conditions. In addition, 3 and 21 day old sugar-fed mosquitoes were given access to a human arm for blood feeding, and within 10 min after taking a full meal, the blood-fed females were used in the trials.

**Figure 1:**
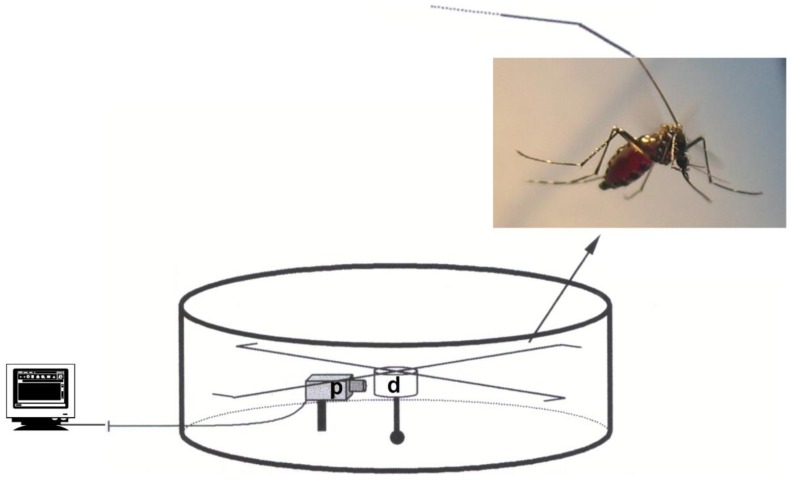
Flight mill (12 cm diameter × 6 cm height) with an aluminium drum (d), four arms of steel wire, and a photointerrupter (p). A female mosquito is attached to the end of one arm with wax on its scutum. The photointerrupter transmits data to a computer.

**Flight performance:** For the flight experiments, only single females (chilled on ice) with similar wing lengths between 3.2 to 3.6 mm (measured from the alula to the tip, including the fringe) were attached to an arm (using hot wax) and flown on flight mills (Figure 1) constructed according to Rowley and colleagues [22]. The circumference of the flight path is 32.7 cm, and the number of revolutions was recorded by a computer at 30 s intervals. Flight experiments usually started around noon and lasted for 16 h at room temperature (24 ± 2 °C) under summer light conditions. From each of several cohorts, 20 females were staged for each of the different nutritional conditions and ages. Females were subjected to only one flight trial, and data from females flying <0.5 km were not included for analysis, because poor flights most often indicated faulty mounting onto the flight arm. After each flight trial, the following data were calculated for each female: total distance flown during the test period, average active flight time (*i.e.*, sum active flight time in the 30 s interval), flight speed, and temporal flight/rest pattern (*i.e.*, segments of continuous flights or erratic flight pulses). The flight data was analyzed with PASW Statistics 18 software [23] using ANOVA, Tukey-HSD (alpha = 0.05) and error terms were given with ± STDEV.

## 3. Results

**Figure 2:**
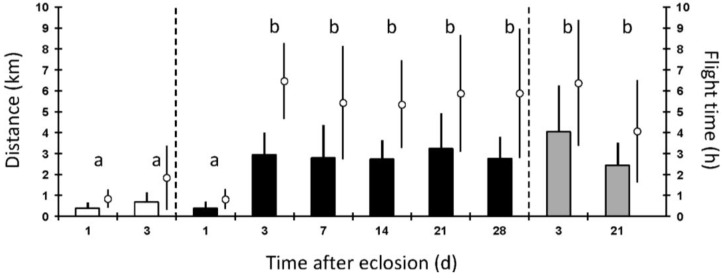
Total flight distances (bars) and average flight times (circles above the bars) for starved (water only; white bars), sugar-fed (10% fructose solution; black bars), and blood-fed (sugar-fed with a blood meal immediately before the experiment; grey bars) females of different ages. Data shown are means (±STDEV) from n = 14–19 for each treatment recorded on flight mills (Oneway Anova, Tukey-HDS, *p* ≤ 0.05; for distance and flight time).

When tested on the first day post eclosion, starved and sugar-fed *Ae. albopictus* females flew an average distance of 0.4 ± 0.3 km/16 h (Figure 2). After 3 days of starvation, females covered an average distance of 0.7 ± 0.5 km/16 h, which is not significantly different from that of 1 day old starved females. By day 3, sugar-fed females flew a much greater distance of 2.7 ± 1.2 km/16 h, and females with continuous access to the sugar solution maintained this flight ability for up to 4 weeks (overall average distance of 2.6 ± 1.4 km/16 h; Figure 2). Three day old blood-fed females flew 3.8 ± 2.3 km/16 h (Figure 2), which is not significantly different than the distance flown by sugar-fed females of the same age. The flight of 3 week old blood-fed females was again not significantly different from that of similarly aged, sugar-fed females.

The average flight time for the different groups of experimental females is presented in Figure 2, and the same trends described above are evident. One day old females starved or sugar-fed spent a similar time flying (0.85 ± 0.44 h and 0.82 ± 0.48 h, respectively). The flight times of 3 day old sugar- or blood-fed females did not statistically differ (5.84 ± 2.20 h and 6.03 ± 3.22 h, respectively), but the times were significantly longer (*p* ≤ 0.05) than those of starved females (1.85 ± 1.53 h). At 3 weeks post eclosion, both sugar- and blood-fed females had similar flight times as that of day 3 females (*p* > 0.5). Blood-fed females flew an average of 3.84 ± 2.50 h, which was less than that of sugar-fed sisters (5.06 ± 3.05 h), though not significantly different. Within the three feeding treatments, the mean flight speed of differently aged mosquitoes was similar. However, starved and sugar-fed females flew significantly slower than their blood-fed sisters (0.43 ± 0.17 km/h, 0.50 ± 0.18 km/h, and 0.68 ± 0.23 km/h, respectively).

The average of maximal continuous flight segments per flight trial of starved females (0.38 ± 0.15 h) was significantly different (*p* ≤ 0.05) from that of sugar- and blood-fed females (2.14 ± 0.69 h and 3.17 ± 0.82 h, respectively). The data for this parameter were not significantly different for the two latter groups. Under starvation, most females (83%) performed maximal flight segments that lasted no longer than 30 min (Table 1). Only two starved females (11%) showed flight segments of more than 30 min but less than 1 h, and the best flying starved female had a continuous flight segment of 1.28 h. A distinctly different pattern was obvious for the sugar- and the blood-fed females, which generally flew continuously for longer than 1 h (76 and 88%, respectively), but such flights were beyond the capability of all but one starved female.

**Figure 3:**
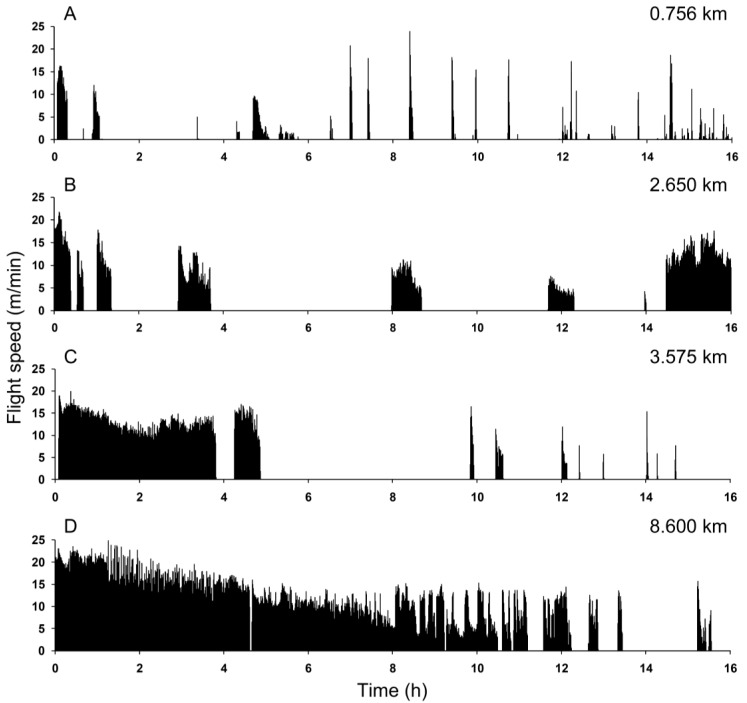
Representative flight profiles for individual female *Ae. albopictus* (3 days old) over 16 h with total distance given at the top right, and recorded flight speed given as m/min on the X axis. (**A**) Starved (access to water only), (**B**) sugar-fed (10% fructose solution), and (**C**) blood-fed immediately before the experiment (prior access to 10% fructose solution). (**D**) Exceptional flight of a blood-fed female.

Representative flight recordings for individual 3 day old starved, sugar- or blood-fed females are presented in Figure 3. After mounting, females immediately started to fly. The duration of the initial flight was less than half an hour for most (72%) of the starved females, and thereafter, they took short flight spurts and needed longer resting periods (Figure 3A). Initial non-stop flights lasted more than 1 h for 37% of sugar-fed females, and then, they took fewer but longer continuous flights (Figure 3B), as compared with their starved sisters (Table 1). Blood-fed females were capable of long continuous flights (>3.5 h) with a majority (82%) flying more than 1 h non-stop for the initial flight (Figure 3C). One remarkably energetic blood-fed female flew nearly 11 h without any substantial interruption (Figure 3D).

**Table 1:** Percentage of maximal continuous flight segments for female *Ae. albopictus* (day 3 post eclosion) in different nutritional states.

	Maximal continuous flights (h)				
	<0.5	0.5–1	1–2	2–4	>4
Feeding condition ^1^ (number of fliers)	%				
Starved (18)	83	11	6	0	0
Sugar-fed (16)	6	19	25	37	13
Blood-fed (17)	6	6	12	41	35

^1^ Starved: access to water only; sugar-fed: access to 10% fructose solution; blood-fed: sugar-fed females that took a blood meal immediately before the trial.

## 4. Discussion and Conclusions

This is the first study to evaluate the flight performance of female *Ae. albopictus* using a flight mill system and to determine the effects of different nutritional states. Females starved (access to water only) up to three days after emergence exhibited short and fast flight bursts that may indicate searches for natural sugar sources needed to sustain flight after eclosion. However, females given immediate access to sugar did not exhibit increased flight performance at day 1, as compared to starved mosquitoes, but females given sugar solution for 3 days flew a significantly greater distance. This result may indicate that during the first day after eclosion the ingested sugar was stored as glycogen and lipid and later metabolized for flight fuel [21]. As well, the ingested sugar and its metabolites may provide energy for the maturation of the “mosquito flight machinery” and other organs [19,24], which restricts the flight range during the first day after eclosion. Further, the uptake of sugar by *Ae. albopictus* was shown to be crucial for increased longevity, whereas blood fed and starved females had similar and shorter life-spans [25]. In contrast, the survival and strong flight performance of the highly anthropophilic African malaria vector *Anopheles gambiae*, a facultative sugar feeder, was not enhanced or dependent on ingested sugar [16,26].

*Aedes albopictus* females provided with sugar and then a blood meal did not show a different flight performance (time and distance flown) than those given sugar only; similar results were observed for *Ae. aegypti* [17]. However, the flight speed of *Ae. albopictus* was affected by nutritional state, *i.e.*, blood-fed females flew faster than the starved and the sugar-fed ones. This trend suggests blood-fed mosquitoes have longer continuous flights, and therefore less low-speed initiations and terminations of flights. The natural behaviour of mosquitoes after a blood meal is to search for a resting place to digest the blood and complete egg development. Approximately 35 to 50% of the digested blood metabolites are used for egg production in this mosquito species [21], and some of the remaining metabolites may be used as flight fuel. As shown here, blood-fed females are capable of long continuous flights, e.g., 35% exhibited flights lasting longer than 4 h (Table 1), and most flew longer than 1 h immediately after mounting onto the flight mill. In this situation, females appear to commence searching for a resting place but continuously fly when forced to be air-borne tethered on the flight mill system. How this affects blood digestion, apportionment of metabolites (sugars and/or lipids), or egg development is yet to be resolved. However, it shows clearly that the blood does not shut down the female’s ability to fly and escape a predator or other disruption.

Comparative experiments with similarly aged and nourished mosquitoes have been performed with *Ae. vexans*, *Ae. aegypti*, *An. gambiae* and *An. atroparvus* [16,17,18]. In general, *Aedes* spp*.* females are stronger fliers (mean distance of around 7 km) than the *Anopheles* spp*.*, which flew about half this distance. These experiments were done in the same laboratory with the same flight mill equipment. In our study, sugar-fed *Ae. albopictus* females flew an average of 3 km, which is comparable to that of the *Anopheles* spp., and this discrepancy in *Aedes* spp*.* performance may reflect different rearing conditions (e.g., larval diet, climatic environment, et cetera) and the newness of the flight mills. Because *Ae. albopictus* has largely replaced *Ae. aegypti* in the southeastern U.S., their developmental and behavioural characteristics are often compared [27,28,29]. One of our hypotheses for this phenomenon was that *Ae. albopictus* is a stronger flier in comparison to *Ae. aegypti*, thus able to disperse further. Our results suggest the opposite in that *Ae. albopictus* females are weaker fliers in comparison to *Ae. aegypti* females, which fly on average 4.5 km in 16 h flight mill trials [17] and up to 14 km when flown to exhaustion [30]. These parameters were determined in different laboratories and conditions, and a direct comparison of their flight performance should be done in the same laboratory under identical conditions, as accomplished in a recent study of the flight performance of wild *vs*. transgenic *Ae. aegypti* males [20].

The flight behaviour of *Ae. albopictus* has been investigated in the field. Mark-release-recapture experiments revealed the mean distance travelled by females (or males) was approximately 200 m, with maximal flight ranges between 50 to 325 m, as determined 4 to 23 days after release [31,32,33]. Another approach was to feed females with rubidium so that it would be incorporated into eggs and readily detected at oviposition sites during one gonotrophic cycle [34]. The suitability of this approach was proven in a pioneering field study with *Ae. aegypti* which flew similar distances compared to *Ae. albopictus* [35]. Studies with *Ae. albopictus* revealed that within one gonotrophic cycle (4 and 6 days in duration) females always reached the periphery of the inspected area. The most distant point was 280 m or 800 m from the release site, respectively [27,29], thus indicating that females likely disperse beyond this range. In addition, it was shown that *Ae. albopictus* readily disperses vertically, e.g., rubidium was detected in eggs on the ground floor as well as 60 m above ground after the release of marked females in the middle of the building [29]. Notably, guidelines for focal vector control of Dengue postulate that most of the relevant vector mosquitoes, including *Ae. albopictus*, stay within 100 m of the place where they emerged [36], but field studies [29,35] and our laboratory study show that *Ae. albopictus* females can disperse over a much greater distance—up to several kilometres within a few days, thus encountering a greater number of hosts and breeding sites. Future studies may require both flight mill and field trials with laboratory-reared and wild *Ae. albopictus* to better estimate their ability to disperse and survive as pathogen vectors.
